# Improving Sparsity and Modularity of High-Order Functional Connectivity Networks for MCI and ASD Identification

**DOI:** 10.3389/fnins.2018.00959

**Published:** 2018-12-18

**Authors:** Yueying Zhou, Limei Zhang, Shenghua Teng, Lishan Qiao, Dinggang Shen

**Affiliations:** ^1^School of Mathematics Science, Liaocheng University, Liaocheng, China; ^2^College of Electronic and Information Engineering, Shandong University of Science and Technology, Qingdao, China; ^3^Department of Radiology and BRIC, University of North Carolina at Chapel Hill, Chapel Hill, NC, United States; ^4^Department of Brain and Cognitive Engineering, Korea University, Seoul, South Korea

**Keywords:** high-order correlation, functional connectivity network, dynamic network, modularity, mild cognitive impairment, autism spectrum disorder

## Abstract

High-order correlation has recently been proposed to model brain functional connectivity network (FCN) for identifying neurological disorders, such as mild cognitive impairment (MCI) and autism spectrum disorder (ASD). In practice, the high-order FCN (HoFCN) can be derived from multiple low-order FCNs that are estimated separately in a series of sliding windows, and thus it in fact provides a way of integrating dynamic information encoded in a sequence of low-order FCNs. However, the estimation of low-order FCN may be unreliable due to the fact that the use of limited volumes/samples in a sliding window can significantly reduce the statistical power, which in turn affects the reliability of the resulted HoFCN. To address this issue, we propose to enhance HoFCN based on a regularized learning framework. More specifically, we first calculate an initial HoFCN using a recently developed method based on maximum likelihood estimation. Then, we learn an optimal neighborhood network of the initially estimated HoFCN with sparsity and modularity priors as regularizers. Finally, based on the improved HoFCNs, we conduct experiments to identify MCI and ASD patients from their corresponding normal controls. Experimental results show that the proposed methods outperform the baseline methods, and the improved HoFCNs with modularity prior consistently achieve the best performance.

## Introduction

Currently, resting state functional magnetic resonance imaging (rs-fMRI), which treats blood oxygen level dependent (BOLD) signals as indirect measures of neural activities, has been widely used in the fields of medicine and neuroscience (Liu et al., 2008; van den Heuvel and Hulshoff Pol, 2010; Liu F. et al., 2013). Based on rs-fMRI, the study of functional connectivity network (FCN) has become a prevalent way to understand brain working mechanism and provide promising biomarkers for diagnosing neural or mental disorders (Bullmore and Sporns, 2009; Fornito et al., 2015; Liu et al., 2017), such as autism spectrum disorder (ASD) (Weng et al., 2010; Wee et al., 2016), Alzheimer's disease (AD) (Wang et al., 2006; Sanz-Arigita et al., 2010), mild cognitive impairment (MCI) (Rombouts et al., 2005; Qiao et al., 2016), major depressive disorder (Greicius et al., 2007; Cullen et al., 2014), schizophrenia (Jafri et al., 2008), and social anxiety disorder (Liu et al., 2015).

To date, researchers have developed many FCN estimation methods (Smith et al., 2011) from the simplest Pearson's correlation (PC) to the most complex dynamic causal modeling. In this paper, we mainly focus on the correlation-based methods due to their relative simplicity and reliability (Smith et al., 2013). Further, we suppose each node of the FCN corresponds to a brain region or a spatial region of interest (ROI) that has been well-defined according to a certain brain atlas, and each “edge” of the FCN corresponds to the relationship between BOLD signals that are extracted from the associated ROIs.

In the conventional correlation-based FCN models, PC is the most popular one for calculating the relationship between ROIs (Smith et al., 2013), but it only captures full correlation without removing the confounding effect from other ROIs. To ease this issue, partial correlations have been employed in calculating the relationship between ROIs for FCN construction (Marrelec et al., 2006). However, the estimation of partial correlation involves the calculation of an inverse covariance matrix, usually resulting in an ill-posed problem. Therefore, many studies adopted a regularizer in the model for more reliable partial correlation estimation (Friedman et al., 2008; Huang et al., 2009; Varoquaux et al., 2010). In fact, the regularizer also plays a role in encoding priors for FCN construction. For example, *l*_1_-norm regularizer is commonly used for encoding sparsity prior of FCN (Lee et al., 2011), and also trace norm regularizer is used for low-rank prior (Liu G. et al., 2013; Qiao et al., 2016). In summary, most of the correlation-based FCN models can be formulated by a matrix-regularized learning framework, where different data fitting terms and regularized terms are combined for estimating FCNs. Please see Table 1 in section Related Methods for more details.

**Table 1:** Correlation-based FCN estimation methods under a matrix-regularized learning framework, where ||·||_*F*_, ||·||_1_, $||\cdot |{|}_{{}^{*}},$ and ||·||_*q*, 1_ denote F-norm, *l*_1_-norm, trace norm and *l*_*q*, 1_-norm of a matrix, respectively.

**Method**	**Data fitting term**	**Regularized term**
PC (Biswal et al., 1995)	$||W-X^{T}X|{|}_{F}^{2}$	N/A
PC_sparsity_ (Li et al., 2017)	$||W-X^{T}X|{|}_{F}^{2}$	||*W*||_1_
PC_scale−free_ (Li et al., 2017)	$||W-X^{T}X|{|}_{F}^{2}$	$\overset{n}{\underset{i,j=1}{\sum }}\gamma_{ij}|W_{ij}|$
SR (Lee et al., 2011)	$||X-XW|{|}_{F}^{2}$	||*W*||_1_
LR (Qiao et al., 2016)	$||X-XW|{|}_{F}^{2}$	||*W*||_*_
SLR (Qiao et al., 2016)	$||X-XW|{|}_{F}^{2}$	_λ_1_||*W*||1_+_λ_2_||*W*||*_
SGR (Wee et al., 2014)	$||X-XW|{|}_{F}^{2}$	_λ_1_||*W*||1_+_λ_2_||*W*||*q*, 1_
WSR (Yu et al., 2017)	$||X-XW|{|}_{F}^{2}$	||*C* ⊙*W*||_1_
WSGR (Yu et al., 2017)	$||X-XW|{|}_{F}^{2}$	_λ_1_||*C* ⊙*W*||1_+_λ_2_||*W*||*q*, 1_

Compared with the aforementioned FCN estimation methods based on low-order correlations, a novel concept of high-order correlation or high-order FCN (HoFCN) has been recently proposed (Chen et al., 2016; Zhang et al., 2016) for measuring more complex interaction information in the brain. Different from the low-order FCN that models the dependency between ROIs, HoFCN aims to capture relationships among different edges. To put it simply, we can consider the ecological chain (network) as an analogy, where the species are regarded as nodes and the ecological chains are the edges between the nodes. In such a way, ecological chains represent the low-order connection information of this network. However, there may exist some relationships among different ecological chains, and the flourishment or destruction of an ecological chain may affect another ecological chain. Therefore, we expect the relationships between edges can provide higher-order information in modeling FCN, compared with the low-order methods that only measure the relationship between nodes.

To date, several studies have suggested that HoFCNs can provide some useful and complementary information for disorder identification (Macke et al., 2011; Plis et al., 2014; Chen et al., 2016; Zhang et al., 2017). For instance, Plis et al. investigated the nonlinear interactions among brain regions in schizophrenia based on mutual information (Plis et al., 2014). Chen et al. proposed to estimate HoFCNs via correlation's correlation, and use a clustering strategy to reduce the dimensionality for efficient computation (Chen et al., 2016, 2017). Guo et al. constructed HoFCN based on minimum spanning tree and applied it for AD classification (Guo et al., 2017). Zhang et al. proposed the hybrid HoFCN for MCI identification based on the linear combination of low- and high-order FCNs (Zhang et al., 2017). Zhao et al. developed the multi-level HoFCN mainly based on PC and applied it for ASD diagnosis (Zhao et al., 2018).

Different from the heuristic methods for defining HoFCNs, Zhou et al. recently proposed to estimate HoFCN based on a rigorous probabilistic framework (Zhou et al., 2018), where a set of low-order correlation matrices (or low-order FCNs) are first calculated separately in sliding windows, and then these correlation matrices are considered as samples for achieving HoFCN by maximum likelihood estimation (MLE). Such a framework *not only* gives a clear theoretical explanation of HoFCN, *but also* provides a way of integrating dynamic information encoded in a sequence of low-order FCNs. However, the initially estimated low-order FCNs may be unreliable, since the use of limited volumes/samples in each sliding window will significantly reduce the statistical power. As such, the derived HoFCN may contain noisy connections inheriting from the low-order counterparts. To deal with this problem, we propose to improve the HoFCNs based on a regularized learning framework. To be specific, we adopt a two-step learning strategy. First, an initial HoFCN is estimated using the MLE method as proposed in Zhou et al. (2018). Then, an optimal neighborhood network of the initially estimated HoFCN is learnt to meet the sparsity and modularity regularizers, aiming, respectively to remove possible noisy connections and encode more informative structure (i.e., modularity) of the network. To verify the effectiveness of our proposed method, we apply the improved HoFCNs to identify subjects with MCI and ASD from normal controls (NCs), respectively. The experimental results show that our proposed methods outperform the baseline methods, and the improved HoFCN with modularity prior consistently achieves the best performance.

The rest of this paper is organized as follows. In section Related Methods, we introduce the correlation-based low-order and high-order FCN modeling methods. In section The Proposed Method, we propose our HoFCN learning strategy, including the motivation, model and algorithm. In section Experiments and Results, we evaluate our proposed method with applications to MCI and ASD identification. In section Discussions, we discuss our findings and several aspects that affect the final performance. Then, we conclude the paper in section Conclusion.

## Related Methods

In this section, we first summarize the existing correlation-based FCN methods into a matrix-regularized learning framework in Table 1. Then, we specifically describe several representative FCN estimation methods, including PC (Biswal et al., 1995), sparse representation (SR) (Lee et al., 2011) and the MLE-based HoFCN estimation (Zhou et al., 2018).

### Pearson's Correlation

As the most popular and simplest method to estimate FCN, PC with its mathematical expression is defined as follows:

(1) $$\begin{array}{r} W_{ij}=\frac{{\left(x_{i}-{\bar{x}}_{i}\right)}^{T}\left(x_{j}-{\bar{x}}_{j}\right)}{\sqrt{{\left(x_{i}-{\bar{x}}_{i}\right)}^{T}\left(x_{i}-{\bar{x}}_{i}\right)}\sqrt{{\left(x_{j}-{\bar{x}}_{j}\right)}^{T}\left(x_{j}-{\bar{x}}_{j}\right)}} \end{array}$$

where${\;x}_{i}\in R^{V},\;i=1,2,\cdots \;,P$, is the extracted time series from the *i*th ROI, *V* is the number of temporal image volumes, *P* is the total number of ROIs, ${\bar{x}}_{i}\in R^{V}\;$is the mean of *x*_*i*_, and *W*_*i, j*_ is the correlation weight between the *i*th and *j*th ROIs. Without loss of generality, we suppose *x*_*i*_is centralized by${\;x}_{i}-{\bar{x}}_{i}\;$and normalized by $\sqrt{{\left(x_{i}-{\bar{x}}_{i}\right)}^{T}\left(x_{i}-{\bar{x}}_{i}\right)}\;$, and thus we can express PC as $W_{ij}={x_{i}}^{T}x_{j}$, or, its equivalent matrix form

(2) $$\begin{array}{r} W=X^{T}X, \end{array}$$

where $X=\left[x_{1},x_{2},\cdots \;,x_{P}\right]\in R^{V\times P}$ is the rs-fMRI data matrix.

In practice, we can treat Equation (2) as the solution of the following optimization problem

(3) $$\begin{array}{r} {min}_{W}||W-X^{T}X|{|}_{F}^{2}, \end{array}$$

where ||·||_*F*_ represents F-norm of a matrix. In this way, we can put PC into the matrix-regularized learning framework reported in Table 1 for a unified understanding.

### Sparse Representation

SR is one of commonly-used methods for calculating (regularized) partial correlation. The mathematical model of SR is given as follows:

(4) $$\begin{array}{r} {min}_{W}\sum_{i=1}^{n}||x_{i}-\sum_{j\neq i}W_{ij}x_{j}|{|}^{2}+\lambda \sum_{j\neq i}|W_{ij}|. \end{array}$$

Similar to PC, it can be rewritten as the following matrix form,

(5) $$\begin{array}{r} min_{W}||X-XW|{|}_{F}^{2}+{\lambda ||W||}_{1},\;s.t.\;W_{ii}=0,\;\forall i=1,\cdots \;,P, \end{array}$$

where ||·||_*F*_ and ||·||_1_ denote F-norm and *l*_1_-norm of the matrix, respectively. The constraint *W*_*ii*_ = 0 plays a role in removing *x*_*i*_ from *X* to avoid trivial solution.

Based on the idea of sparsity, many extended SR methods have been developed for constructing FCNs, including sparse group representation (Wee et al., 2014), weighted sparse representation (Yu et al., 2017), weighted sparse group representation (Yu et al., 2017), sparse low-rank representation (Qiao et al., 2016) and sparse PC (Li et al., 2017), to name a few. Most of these methods can be unified in the matrix-regularized leaning framework as shown in Table 1.

### MLE-Based HoFCN Estimation

As discussed in section Introduction, many HoFCN estimation methods have been proposed in recent years. Here, we only review the MLE-based method (Zhou et al., 2018) due to its clear probabilistic explanation, and shortly we will use this method (named HoFCN_MLE_) as a baseline for developing our approach.

The HoFCN_MLE_ method includes two main steps. First, a set of low-order FCNs are estimated in a series of sliding windows. Then, the resulted low-order FCNs are used as samples for estimating HoFCN by MLE with an assumption that the low-order FCN samples follow a matrix-variant normal distribution (Zhang and Schneider, 2010). As a result, the HoFCN, Ω, can be achieved by the following iteration formula (generally, with the identity matrix *I* as an initial estimation of Ω).

(6) $$\begin{array}{r} \Omega =\frac{1}{KP}\sum_{k=1}^{K}\left(W_{k}-M\right)\Omega^{-1}{\left(W_{k}-M\right)}^{T} \end{array}$$

where *W*_*k*_ is the *k*th low-order FCN associated with the *k*th sliding window, and $M=\frac{1}{K}\overset{K}{\underset{k=1}{\sum }}W_{k}$ is the mean of all the low-order FCNs. Please refer to Zhou et al. (2018) for details of the theoretical formulation and probabilistic explanation.

## The Proposed Method

### Motivation

As discussed earlier, despite its empirical effectiveness in identifying neuropsychiatric disorders, the typically estimated HoFCN may contain some noisy connections that inherit from the low-order FCNs. In general, the weak connections in HoFCN are removed according to a given threshold, prior to the statistical analysis or classification. However, the thresholding scheme is heuristic and only consider the sparsity aspect of FCN. Therefore, in this section we develop a more flexible approach for improving HoFCN based on the matrix-regularized learning framework, *not only* aiming to reduce noisy connections of HoFCN, *but also* introduce more informative structures (i.e., modularity) than just sparsity into HoFCN. In particular, we will consider both sparsity and modularity as the priors of HoFCN, due to their well-accepted neuroscientific basis (Sporns, 2011; Sporns and Betzel, 2016). To our best knowledge, this is the first work to employ the modularity prior in HoFCN estimation.

### Model: Learning Neighborhood Networks With Regularizers

For reaching the above goals, we adopt a simple two-step learning strategy. First, we obtain an initial estimation of HoFCN (denoted by Ω_0_) based on the MLE method as described in section MLE-based HoFCN Estimation. Second, we learn an optimal neighborhood network of the initially estimated HoFCN Ω_0_ with sparsity and modularity priors, respectively, as regularizers of the objective function.

More specifically, sparsity can usually be encoded by *l*_1_-norm regularizer (Lee et al., 2011), and thus the optimal neighborhood network of HoFCN with *sparsity* prior (named **S-HoFCN**) can be achieved as follows:

(7) $$\begin{array}{r} \underset{\Omega }{\min}||\Omega -\Omega_{0}|{|}_{F}^{2}+{\mu ||\;\Omega ||}_{1}, \end{array}$$

where Ω_0_ is the initially estimated HoFCN by MLE method, Ω is the improved HoFCN that needs to be sparse and simultaneously keep as the spatial neighbor of Ω_0_, and μ is the regularized parameter for controlling the balance between the sparsity of Ω and its distance from Ω_0_.

Furthermore, modularity means that some group structures exist in the network, where the nodes within a group are densely connected, while the nodes between groups are sparsely connected (Sporns and Betzel, 2016). Notably, it has been proved that the modularity of a network can be described by a combination of trace (nuclear) norm and *l*_1_-norm under mild conditions (Liu G. et al., 2013; Qiao et al., 2016). Therefore, we optimize the neighborhood network of HoFCN with *modularity* prior (named **M-HoFCN**), as follows,

(8) $$\begin{array}{r} {\text{min}}_{\Omega }||\;\Omega -\Omega_{0}|{|}_{F}^{2}+{\mu_{1}||\;\Omega ||}_{1}+{\mu_{2}||\Omega ||}_{{}^{*}}, \end{array}$$

where μ_1_ and μ_2_ are regularized parameters used to control the balance between the three terms in the optimization problem. Specially, when μ_2_ = 0, Equation (8) reduces to S-HoFCN as given in Equation (7), meaning that the sparsity is a necessary but not sufficient condition for modularity. Note that the proposed models in both Equations (7, 8) are also fit for matrix-regularized learning framework described in Table 1.

### Algorithm

Here, we only give the optimization algorithm for solving M-HoFCN, since S-HoFCN is a special case of M-HoFCN. Note that the objective function in Equation (8) is convex, but the *l*_1_-norm and trace norm are both indifferentiable. To address this kind of optimization problem, a number of algorithms have been developed in the machine learning community (Tomioka and Sugiyama, 2009; Richard et al., 2012; Zhuang et al., 2012; Oymak et al., 2014). We choose the proximal method (Combettes and Pesquet, 2009; Bertsekas, 2012) to solve Equation (8), due to its simplicity and efficiency.

In particular, we first consider the data fitting term $f\left(\Omega \right)=||\;\Omega -\Omega_{0}|{|}_{F}^{2}$ in Equation (8). Since it is differentiable, we can calculate its gradient with respect to Ω, and get ∇*f*(Ω) = 2(Ω−Ω_0_). As a result, we have the gradient descent step as follows,

(9) $$\begin{array}{r} \Omega_{k}=\Omega_{k-1}-\alpha_{k}\cdot \nabla f\left(\Omega_{k-1}\right), \end{array}$$

where α_*k*_ is the step size.

Then, according to the definition of proximal operator (Combettes and Pesquet, 2009), the proximal operator of *l*_1_-norm (i.e., _μ_1_|| Ω||1_) is given as follows,

(10) $$\begin{array}{r} {\text{prox}}_{\mu_{1}||\cdot |{|}_{1}}\left(\Omega \right)={\left[sgn\left(\Omega_{ij}\right)\times \max\left(abs\left(\Omega_{ij}\right)-\mu_{1}\right),0\right]}_{p\times p}, \end{array}$$

where *sgn*(·) and *abs*(·) are sign and absolute functions, respectively. Equation (10) in fact imposes a soft-threshold operation on the entries of Ω. Similarly, the proximal operator of trace norm ${\mu_{2}||\Omega ||}_{{}^{*}}$ is equivalent to a shrinkage operation on the singular value of Ω (Ji and Ye, 2009), as follows.

(11) $$\begin{array}{l} {\text{prox}}_{\mu_{2}\left\|\cdot \right\|}{}_{*}\left(\Omega \right)=Udiag(\max\left\{\sigma_{1}-\mu_{2},0\right\},\\ \;\cdots ,\;\max\left\{\sigma_{n}-\mu_{2},0\right\})V^{T}, \end{array}$$

where $Udiag\left(\sigma_{1},\cdots \;,\;\sigma_{n}\right)V^{T}$ is the singular value decomposition of matrix Ω.

Finally, to circumvent the case that the current Ω_*k*_ moves out of the “feasible region” regularized by *l*_1_-norm || Ω||_1_ and trace norm $||\Omega |{|}_{{\;}^{*}}$, we use the proximal operations prox_μ_1_||·||_1__ and ${\text{prox}}_{\mu_{2}||\cdot |{|}_{{\;}^{*}}}$ on Ω_*k*_, respectively, as given in Equations (10, 11). Hence, we get a simple algorithm to solve Equation (8) as shown in Table 2.

**Table 2:** Algorithm for solving M-HoFCN in Equation (8).

**Initialize Ω with Ω_0_**
Iterate:
1. Ω←Ω−α·2(Ω−Ω_0_)
2. Ω←prox_μ_1_||·||_1__(Ω) = [*sgn*(_Ω_*ij*_) × max(*abs*(Ω_*ij*_)−μ_1_), 0]*p*×*p*_
3. $\Omega \leftarrow {\text{prox}}_{\mu_{2}||\cdot |{|}_{*}}\left(\Omega \right)=Udiag\left(\max\left\{\sigma_{1}-\mu_{2},0\right\},\cdots \;,\;\max\left\{\sigma_{n}-\mu_{2},0\right\}\right)V^{T}$

## Experiments and Results

In this section, we first evaluate the proposed method by identifying subjects with MCI from NCs based on ADNI dataset (http://adni.loni.ucla.edu), and then conduct an ASD identification task based on ABIDE database (http://fcon_1000.projects.nitrc.org/indi/abide/) for further illustrating the generalization of the proposed method.

### Data Acquisition and Preprocessing

For MCI identification, 137 subjects (including 68 MCIs and 69 NCs) were selected in our study. The subjects were age-matched and scanned by 3.0T Philips scanners. SPM8 (http://www.fil.ion.ucl.ac.uk/spm/) toolbox was used to process the acquired rs-fMRI data. For each subject, the scanning time was 7 min, corresponding to 140 volumes. Subjects with more than 2.5 min of large framewise displacement (FD > 0.5) were excluded before data inclusion. To keep signal stabilization, the first 3 volumes of each subject were also removed, and the remaining volumes were corrected for subsequent analysis. During the scan, a rigid-body transformation was applied to correct head motion, and the subjects with head motion larger than 2 mm or 2° were excluded. The rs-fMRI images were registered to the Montreal Neurological Institute (MNI) space and spatially smoothed by a Gaussian kernel with full width at half maximum of 6 × 6 × 6 mm^3^. To further reduce the influences of nuisance signals, regression of ventricle and white matter signals as well as Friston 24-parameter model (Friston et al., 1996) were also performed. Using Automated Anatomical Labeling (AAL) template (Tzourio-Mazoyer et al., 2002), the pre-processed BOLD signals were divided into 116 ROIs, among which 90 ROIs are in the cerebra and the rest 26 are in the cerebella. For each ROI, prior to FCN estimation, its mean rs-fMRI time series was band-pass filtered from 0.015 to 0.15 Hz. At last, all the mean time series of the whole brain were put into a data matrix *X*∈*R*^137 × 116^.

For ASD identification, we use the same preprocessed dataset as in Wee et al. (2016). Specifically, 92 subjects including 45 ASD patients and 47 typically developing NCs (with ages between 7 and 15 years old) from this dataset are selected. All rs-fMRI images were acquired using a standard echo-planar imaging sequence on a clinical routine 3T Siemens Allegra scanner. During 6 min rs-fMRI scanning procedure, the subjects were required to relax with their eyes focusing on a white fixation cross in the middle of the black background screen projected on a screen. The imaging parameters include the flip angle as 90°, 33 slices, TR/TE as 2000/15 ms with 180 volumes, and 4.0 mm voxel thickness. The fMRI data were preprocessed by SPM8. Specifically, the first 10 rs-fMRI volumes of each subject were discarded. The remaining volumes were calibrated as follows: (1) normalization to MNI space with resolution 3 × 3 × 3 mm^3^; (2) regression of nuisance signals (ventricle, white matter, global signals, and head-motion) with Friston 24-parameter model; (3) band-pass filtering (0.01–0.08 Hz); (4) signal de-trending. After that, the BOLD time series signals were partitioned into 116 ROIs, according to the AAL atlas. At last, we put these time series into a data matrix *X*∈*R*^170 × 116^.

### FCN Construction

With the preprocessed rs-fMRI data, we calculate the improved HoFCNs using the proposed S-HoFCN and M-HoFCN methods, respectively. Moreover, for comparison, we construct FCNs based on the baseline methods including PC, SR and HoFCN_MLE_.

**Figure 1:**
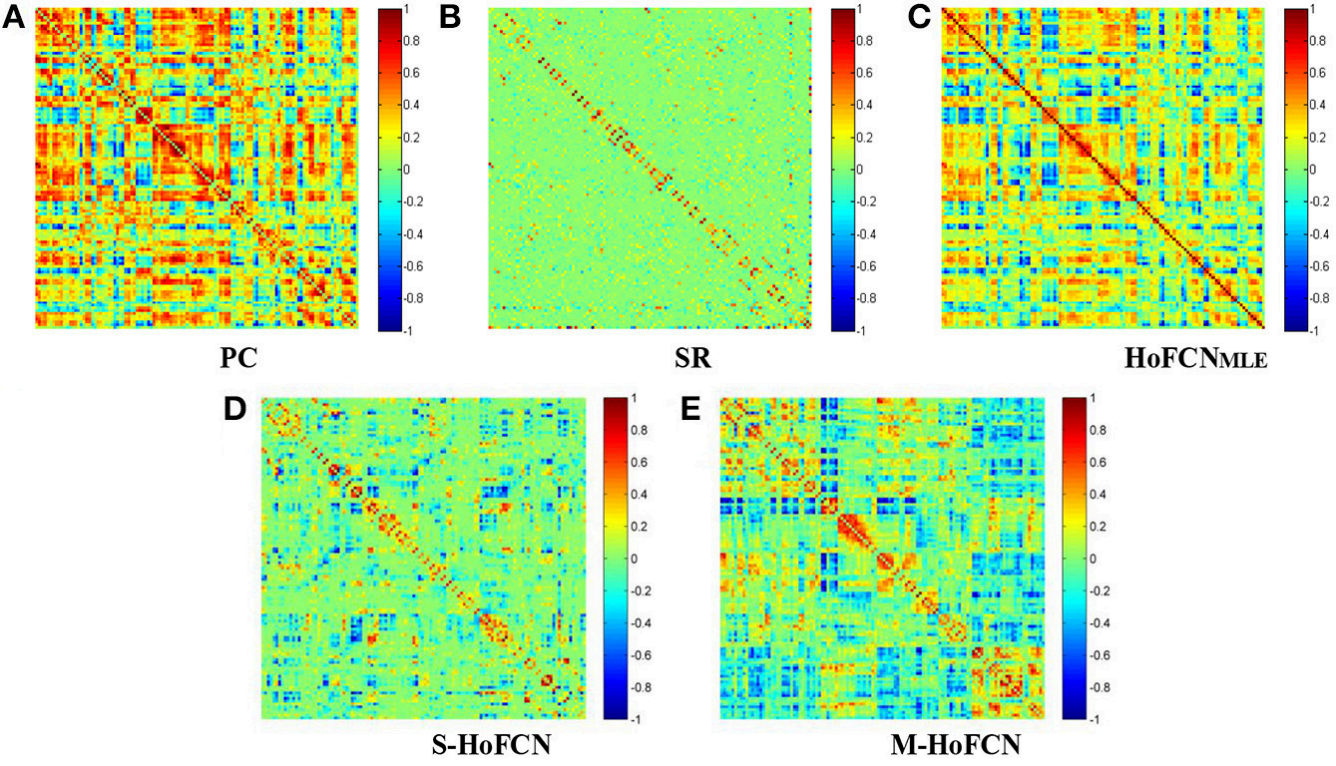
The FBN adjacency matrices constructed by five different methods. **(A)** PC, **(B)** SR, **(C)** HoFCN_MLE_, **(D)** S-HoFCN, and **(E)** M-HoFCN.

In Figure 1, we show the adjacency matrices of a certain FCN constructed by five different methods for MCI identification. The regularized parametric values used in SR, S-HoFCN and M-HoFCN are λ = 2^4^, μ = 2^−3^ and ${\;\mu }_{1}=\;2^{4}$,${\;\mu }_{2}=2^{-3}$, respectively. For the high-order methods, the width of sliding windows is fixed to *N* = 70, and step size *s* = 1. As seen from Figure 1, the networks based on PC and HoFCN_MLE_ are dense, meaning that both low-order and high-order correlations without sparsity constraints may cause some “noisy” connections. In contrast, the networks based on SR, S-HoFCN, and M-HoFCN are sparse due to the introduction of *l*_1_-norm regularizer. Further, we note that M-HoFCN shows clearer modular structures than S-HoFCN, since the combination of *l*_1_-norm and trace norm regularizers has been proved to result in modularity (Qiao et al., 2016). Finally, it is observed that the high-order FCNs shown in Figures 1C–E tend to have more fine-grained modularity than the low-order FCNs shown in Figures 1A,B, which is consistent with the conclusion that the HoFCNs may be able to capture more subtle network structures as discussed in Zhang et al. (2016).

### Feature Selection and Classification

In our experiments, we adopt the edge weights of FCN or HoFCN as features for MCI/ASD identifications. Although the edge weights include the full information of the networks, it typically causes the curse of dimensionality, since the number of feature dimension, i.e., 116 × (116−1)/2 = 6670, is far greater than the sample size (i.e., the number of subjects). To address this problem, we first employ the two-sample *t*-test (*p* = 0.05) to select features before MCI/ASD classifications.

We use the linear support vector machine (SVM) (Chang and Lin, 2011) with default *C* = 1 for conducting the classification task. A leave-one-out cross validation (LOOCV) is adopted in our experiments to estimate the classification performance of different methods. It works in a way that in each run only one of *T* samples (subjects) is adopted for testing while the rest *T*−1 samples are used for training a classifier. Therefore, we can obtain the final performance by averaging results of all the runs.

In general, one or more hyperparameters are involved in the FCN estimation methods. Specifically, for the regularization parameters (i.e., λ in SR, μ in S-HoFCN and μ_1_, μ_2_ in M-HoFCN), we conduct a line or grid search in the range of (2^−5^, 2^−4^, ⋯, 2^0^, ⋯, 2^4^, 2^5^). Note that no such parameters are involved in PC and HoFCN_MLE_. For a fair comparation, we introduce a thresholding parameter into PC and HoFCN_MLE_ to sparsify the initially estimated FCNs by removing the weakly connected edges. To be consistent, we employ 11 threshold values that correspond to different levels of sparsity (1%, 10%, ⋯, 90%, 100%) for PC and HoFCN_MLE_. For example, 10% means that the threshold value is set to remove 90% edges from the FCN, while 100% means all edges are preserved.

To obtain the optimal parameters for each method, we use an inner LOOCV procedure on the training data. Given a parametric value, in the current *T*−1 training samples for the classification task, we use *T*−2 samples to select features (*t*-test with *p* = 0.05) and train a classifier (SVM with *C* = 1), while the rest one to validate the performance of the trained classifier. Once the best validation performance is achieved by averaging the accuracies of all the inner LOOCV runs, we can determine the optimal value of the parameter for the current training samples. It is worth noting that there are sliding window parameters used in estimating the initial HoFCNs. We will have a detailed discussion about this problem in Section Sensitivity to Network Modeling Parameters.

**Figure 2:**
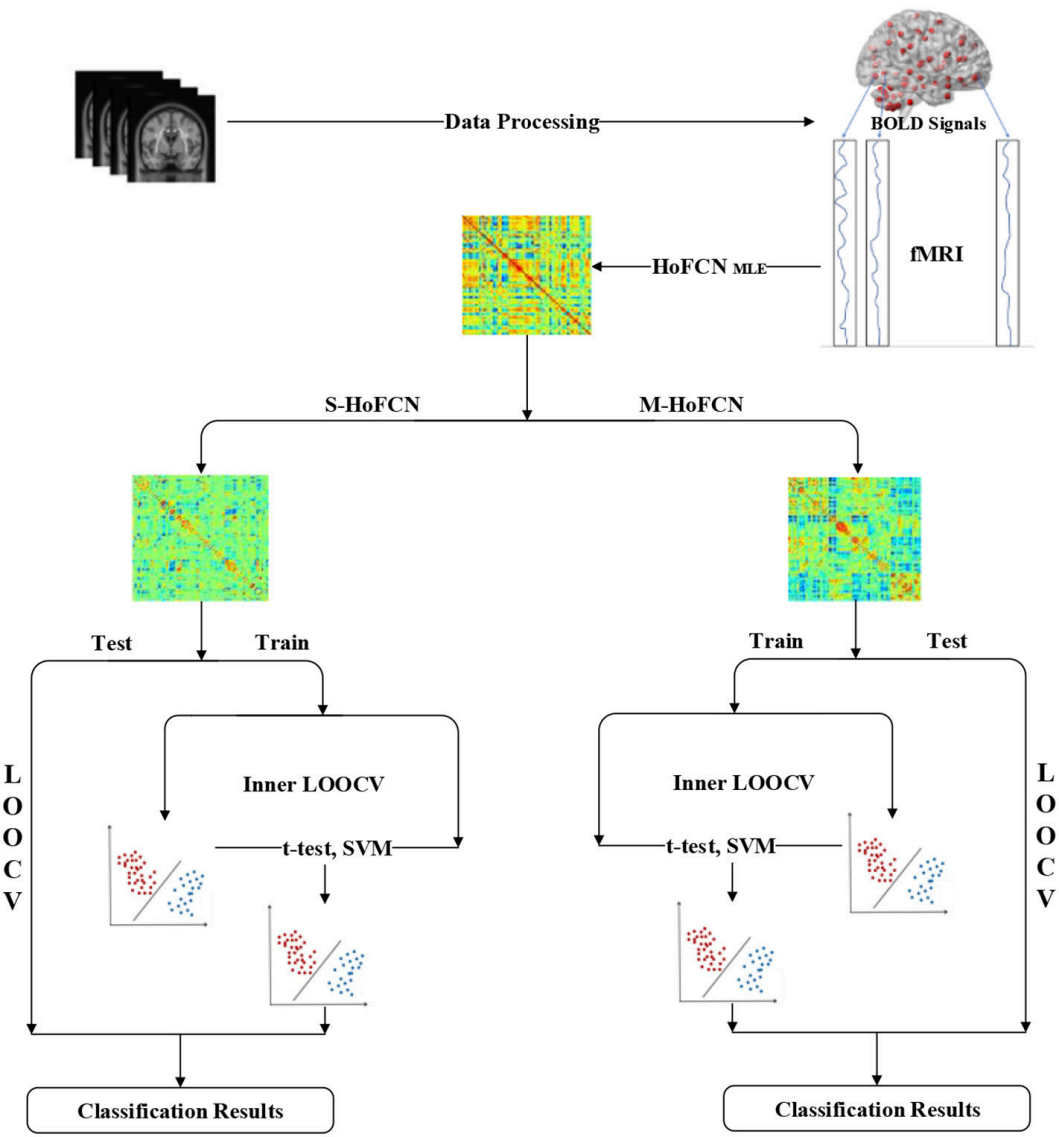
The pipeline of MCI/ASD identifications used in this study.

In Figure 2, we display the pipeline of MCI identification used in our experiments. Based on the preprocessed fMRI data, we first estimate the initial HoFCNs based on the MLE method, and then improve the initially estimated HoFCNs by introducing sparsity and modularity priors, respectively. Finally, we apply the improved HoFCNs to identify patients suffering from MCI or ASD via the LOOCV scheme.

To evaluate the different FCN estimation methods, we adopt accuracy (ACC), sensitivity (SEN) and specificity (SPE) (Sokolova et al., 2006) as performance metrics. Their definitions are given in Table 3, where TP, TN, FP, and FN indicate true positive, true negative, false positive and false negative, respectively. Of note, in this work, we treat MCI/ASD patients as the positive class while the NCs as the negative.

**Table 3:** Definitions of the performance metrics involved in this paper.

**Performance metric**	**Abbreviation**	**Definition**
accuracy	ACC	$\frac{TP+TN}{TP+FP+TN+FN}$
sensitivity	SEN	$\frac{TP}{TP+FN}$
specificity	SPE	$\frac{TN}{TN+FP}$

### Classification Results

In Table 4, we report the classification results of five different methods for MCI classification task. For the three HoFCN methods, we report the best result based on different sizes of sliding windows (*N* = 50, *s* = 1 for HoFCN_MLE_; *N* = 70, *s* = 2 for S-HoFCN; and *N* = 70, *s* = 6 for M-HoFCN). As shown in Table 4, with respect to ACC and SPE, the proposed methods outperform the baseline methods, and they are consistently better than HoFCN_MLE_. Especially for M-HoFCN, it achieves the best performance. Therefore, we argue that the modularity prior is of vital importance in removing the noisy connections and improving the reliability of HoFCNs. However, we note that, in terms of SEN, the proposed methods do not work well. In the next section, we will further investigate this phenomenon.

**Table 4:** The classification results based on five different methods for MCI identification, with *N* = 50, *s* = 1 for HoFCN_MLE_, *N* = 70, *s* = 2 for S-HoFCN, and *N* = 70, *s* = 6 for M-HoFCN.

**Method**	**ACC**	**SEN**	**SPE**
PC	0.7956	0.8824	0.7101
SR	0.7810	0.8088	0.7536
HoFCN_MLE_	0.8248	0.8824	0.7681
S-HoFCN	0.8540	0.8676	0.8406
M-HoFCN	0.8613	0.8382	0.8841

In Table 5, we simply report the best experimental results of ASD identification. For three HoFCN methods, *s* is fixed with 1, while *N* = 90, 110, 70 for HoFCN_MLE_, S-HoFCN, and M-HoFCN, respectively. In terms of ACC, the proposed methods perform better than low-order FCNs, and better than the results reported in Wee et al. (2016). Similar to the results on MCI dataset, the M-HoFCN method also achieves the best performance, meaning that the modularity prior plays an important role in FCN modeling.

**Table 5:** Comparison of the best classification results based on six different methods for ASD identification. Here, we empirically fix *s* = 1, and *N* = 90, 110, 70 for HoFCN_MLE_, S-HoFCN and M-HoFCN, respectively.

**Method**	**ACC**	**SEN**	**SPE**
PC	0.6304	0.6222	0.6383
SR	0.5543	0.6000	0.5106
Wee et al., 2016	0.7070	0.8140	0.6120
HoFCN_MLE_	0.7391	0.8000	0.6809
S-HoFCN	0.7391	0.7778	0.7021
M-HoFCN	0.7500	0.8000	0.7021

## Discussions

### Sensitivity to Network Modeling Parameters

**Figure 3:**
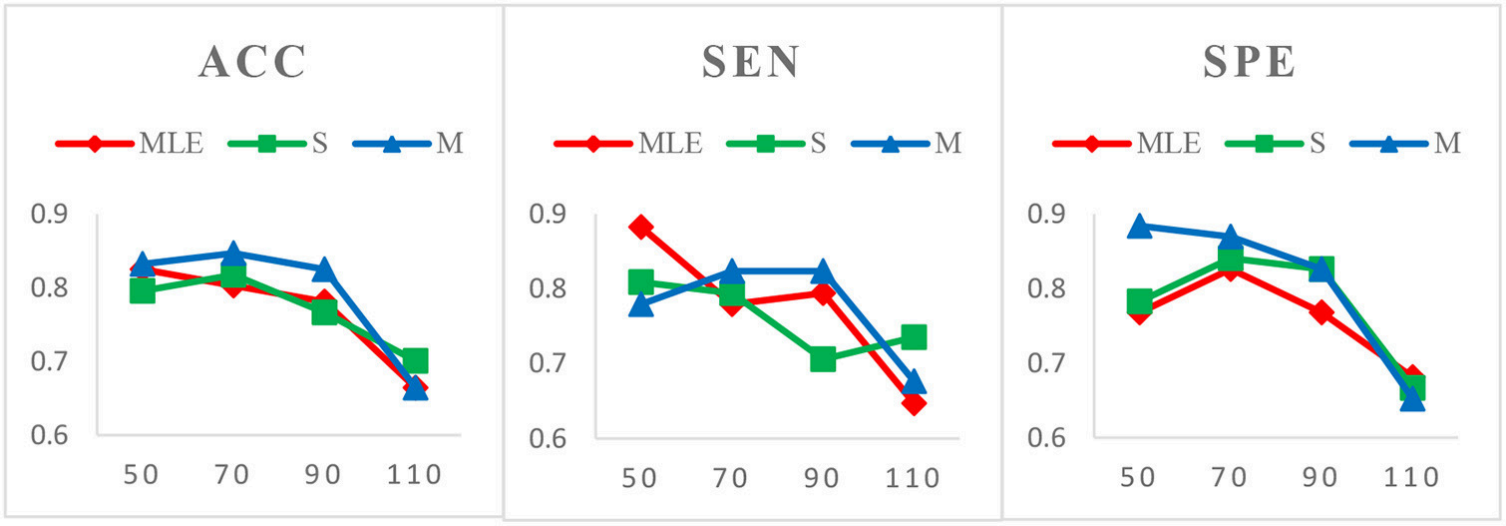
Comparison of classification results based on three HoFCN methods for MCI identification, with varied window widths *N* = 50, 70, 90, 110 and fixed step size *s* = 1. The proposed methods (especially M-HoFCN) are consistently better than HoFCN_MLE_, and they tend to have the best classification performance at *N* = 70.

**Figure 4:**
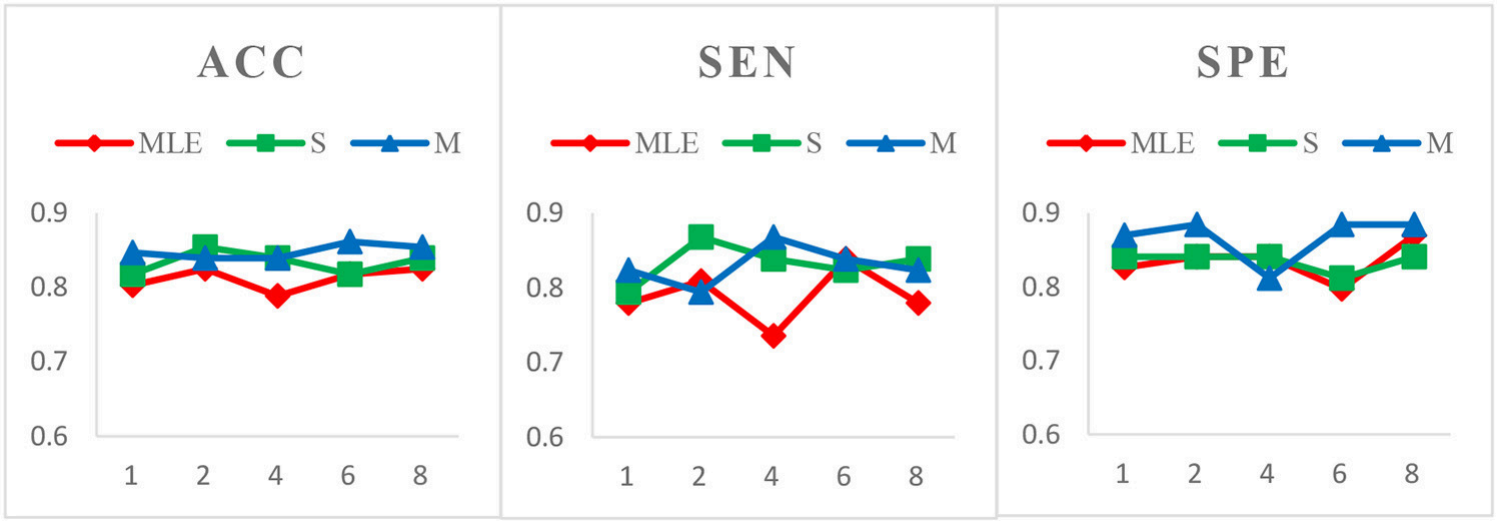
Comparison of classification results based on three HoFCN methods for MCI identification, with different step size *s* = 1, 2, 4, 6, 8 and fixed window width *N* = 70. Our proposed methods have 80% possibilities to perform better than HoFCN_MLE_.

**Figure 5:**
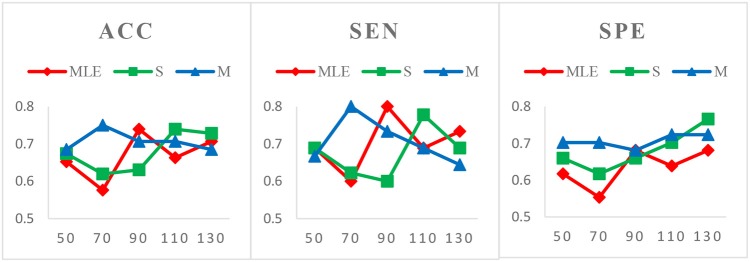
Comparison of classification results based on three HoFCN methods for ASD identification, with varied *N* = 50, 70, 90, 110, 130 and fixed *s* = 1. Note that M-HoFCN achieves the best performance in average.

In this study, the involved parameters can be divided into two groups, i.e., sliding window parameters of HoFCNs and the regularization parameters (or threshold values) in the network estimation models. As discussed earlier, we have selected the optimal regularized parameter via inner LOOCV. In the following, we will discuss the sensitivity to the parameters of sliding windows (i.e., width *N* and step size *s*) of three HoFCN methods (HoFCN_MLE_, S-HoFCN and M-HoFCN). To this end, we conduct experiments for MCI/ASD identifications under three cases:

- *Case 1*: Varied window widths *N* = 50, 70, 90, 110 and fixed step size *s* = 1 for MCI identification. Figure 3 shows the classification results of three HoFCN methods under this case. (Of note, in both Figures 3–5, we simply use MLE, S and M to represent the corresponding HoFCN_MLE_, S-HoFCN and M-HoFCN methods, respectively.) It can be observed that our proposed methods perform better than HoFCN_MLE_ and have the best performance at *N* = 70. For ACC, M-HoFCN has 100% possibilities to outperform HoFCN_MLE_, and 75% possibilities to outperform S-HoFCN. For SEN and SPE, M-HoFCN has 75% possibilities to work better than HoFCN_MLE_. That is, M-HoFCN is the best method for MCI classification. Additionally, we note that the bigger value of *N* tends to result in worse performance, which is consistent with the finding of choosing window sizes in Hindriks et al. (2016).
- *Case 2*: Fixed window width *N* = 70 and varied step sizes *s* = 1, 2, 4, 6, 8 for MCI identification. We choose *N* = 70 since, as shown in Figure 3, the HoFCN methods tend to have the best performance at 70. In this way, we show classification results based on three HoFCN methods in Figure 4. By comparison, for ACC, the proposed methods have 100% possibilities to outperform HoFCN_MLE_, and M-HoFCN has 80% possibilities to outperform S-HoFCN; for SEN and SPE, S-HoFCN and M-HoFCN both have 80% possibilities to perform better than HoFCN_MLE_. As such, our proposed methods at least have 80% possibilities that perform better than HoFCN_MLE_, and M-HoFCN has the best performance at *N* = 7 0, *s* = 6, while S-HoFCN at *N* = 70, *s* = 2. By using the fixed window width *N* = 70, we find that the performances of three HoFCN methods with varied step sizes are relatively stable.
- *Case 3*: Varied window widths *N* = 50, 70, 90, 110, 130 and fixed *s* = 1 for ASD identification. As shown in Figure 5, S-HoFCN has 80% possibilities to work better than HoFCN_MLE_ for ACC and SPE, and 60% for SEN; M-HoFCN has 100% possibilities to outperform HoFCN_MLE_ for SPE, and 60% for ACC and SEN. For different methods, HoFCN_MLE_ gets the best performance at *N* = 90, while S-HoFCN at *N* = 110 and M-HoFCN at *N* = 70. Compared with the performance of three HoFCN methods, we found that M-HoFCN averagely achieves the best performance and it remains more stable than the other two methods. It is consistent with the finding in MCI identification.

### Parcellation of the Brain

We adopt AAL atlas with 116 ROIs as network nodes in this work. To date, there exists different ROI definitions in different brain anatomical/functional templates, such as AAL, Harvard-Oxford (http://www.fmrib.ox.ac.uk/fsl/), Eickhoff-Zilles (Eickhoff et al., 2005) and Automatic Non-linear Imaging Matching and Anatomical Labeling (ANIMAL) (Collins et al., 1995; Wang et al., 2009), etc. As reported in Wang et al. (2009), AAL and ANIMAL templates can lead to significant differences in network topological properties. Craddock et al. also revealed that ROI size had great impact on the network performance analysis, and 200 ROIs can offer better interpretability (Craddock et al., 2012). Therefore, we further conduct experiments for constructing FCNs with 200 ROIs from (Craddock et al., 2012), and take ASD classification as an example for evaluating the influence of different parcellation schemes on the final accuracy. Experimental results show that the proposed M-HoFCN still achieves the best ACC (0.6923), compared with PC (0.6248), SR (0.5167), HoFCN_MLE_ (0.6811), and S-HoFCN (0.6730), which further illustrates the importance of using modularity prior in FCN estimation. Note, however, that the performance of most methods reduces greatly with 200 ROIs. The possible reason is that 200 ROIs, resulting in 200 × (200−1)/2 = 19900 edges, cause the challenge for feature selection or the “curse of dimensionality,” since limited training samples are involved. In the future, we plan to further investigate the influence of differently selected templates on the network properties and the subsequent classification performance.

### Head Motion Artifacts

As we know, the FCN estimation based on rs-fMRI data is sensitive to the head motion (Van Dijk et al., 2012; Power et al., 2014). To eliminate the influence of head motion artifacts, a commonly used method is data scrubbing that removes some volumes based on FD or DVARS (Power et al., 2012). However, in this study, we did not perform scrubbing operation to exclude volumes since dynamic information is necessarily encoded by the sliding window scheme for estimating initial low-order FCNs. The removal of volumes would disrupt the autocorrelation structure of data, which is problematic related to temporal filtering and dynamic information encoding (Janine et al., 2017). In fact, several related studies (Chen et al., 2016, 2017) also did not suggest scrubbing operation due to the same reason. Additionally, the data scrubbing often removes relatively high amount of data, thus reducing the statistical power, and, in practice, how to determine a suitable threshold of FD is still an open problem.

To further study the impact of head motion artifacts on the classification performance, we conduct the network modeling methods without regression of Friston 24-parameters. With fixed parameters such as *N* = 70 and *s* = 1, the experimental ACCs for ASD identification are PC (0.6750), SR (0.6104), HoFCN_MLE_ (0.6511), S-HoFCN (0.6430), and M-HoFCN (0.6458), respectively. Compared with the results in Table 5, we note that most of the methods tend to decrease ACC, meaning that head motion artifacts have a significant influence on the classification performance. In other words, regressing out the head motion artifacts can contribute to achieve better (at least more discriminative) FCNs.

### Top Discriminative Features

**Figure 6:**
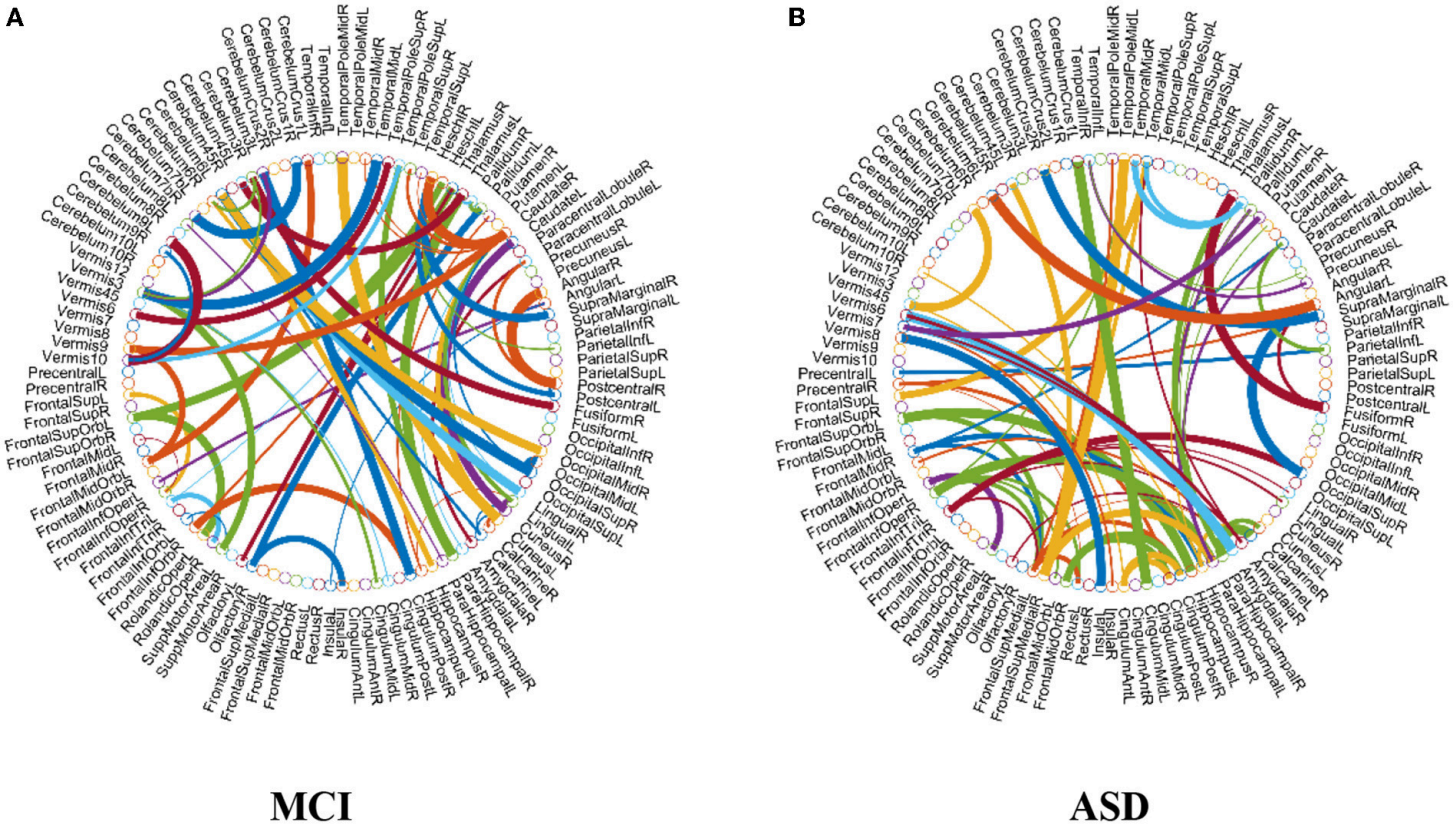
The most discriminative features (network connections) involved in the classification tasks by using *t*-test with *p* < 0.001. Note that the thickness of the arcs is inversely proportional to the corresponding *p*-value for indicating the discriminative power of the features. **(A)** MCI and **(B)** ASD.

As previously mentioned in section Feature Selection and Classification, we adopt the edge weights of estimated FCN as features for classification. Here, we use two-sample *t*-test to re-select the features for MCI and ASD identification problems, respectively, based on the proposed M-HoFCN method, since it achieves the best performance. In particular, after constructing FCNs by M-HoFCN model (*N* = 70, *s* = 6 for MCI; and *N* = 70, *s* = 1 for ASD), we apply *t*-test to select discriminative features in order of their *p*-values (<0.001). In this way, we select 72 and 67 discriminative features for MCI and ASD identification tasks, respectively, and visualize the features in Figure 6. Of note, the thickness of each arc represents the discriminative power that is inversely proportional to the corresponding *p*-value.

From Figure 6A, we found that the top discriminative features (i.e., functional connections) and their corresponding brain regions include right inferior frontal gyrus, bilateral hippocampus, bilateral parahippocampal gyrus, right pallidum, right caudate, left middle temporal gyrus, left cerebellum 6, etc. The findings are partially consistent with previous studies (Wolf et al., 2003; Albert et al., 2011; Solodkin et al., 2013). In particular, the right inferior frontal gyrus (Salvatore et al., 2015), bilateral hippocampus (Chen et al., 2016), bilateral parahippocampal gyrus (Echávarri et al., 2011), right pallidum (Supekar et al., 2008; Albert et al., 2011), right caudate (Albert et al., 2011), left middle temporal gyrus (Kosicek and Hecimovic, 2013; Chen et al., 2016), and left cerebellum 6 (Suk et al., 2015) are all reported as potential biomarkers for MCI or AD identification. However, currently it is an open problem for explaining these FCN-based biomarkers. In the future, we plan to provide further experimental evidences toward the biological explanation of those involved functional connectivity or brain regions.

In terms of the selected features as shown in Figure 6B, brain regions that may contribute to ASD identification in this work include the left precentral gyrus, right middle frontal gyrus, right hippocampus, bilateral parahippocampal gyrus, right amygdala, bilateral putamen, left caudate, bilateral pallidum, and bilateral middle temporal, many of which are widely reported in the previous studies associated with ASD identification (Sparks et al., 2002; Haznedar et al., 2006; Rojas et al., 2006; Toal et al., 2009; Ecker et al., 2010; Qiu et al., 2010).

### Limitations

In this work, we only use PC as a cornerstone in construction of HoFCN due to its simplicity and popularity. However, as we described above, PC can only capture the full correlation, and thus partial correlation-based methods such as SR may be considered in practice. Besides, many researchers have devoted their efforts to FCN estimation methods based on group analysis. For example, Liu et al. proposed a hierarchical Markov random field model to capture both group and subject FCNs simultaneously, which can take within-subject coherence and between-subject consistency of the network label maps into account (Liu et al., 2014); Ghanbari et al. designed a multi-layer graph clustering algorithm to extract hub-networks by non-negative matrix factorization and applied the hubs to characterize population commonalities and subject variations between ASD and typically developing children (Ghanbari et al., 2017); Kam et al. proposed a multiple FCN model using hierarchical clustering and applied it for ASD diagnosis (Kam et al., 2017). These methods provide a different understanding toward brain structure and group/subject analysis, which we can consider in our future studies.

## Conclusion

In this paper, we develop an effective way of improving HoFCN estimation, by learning a neighborhood networks of the initial HoFCN with sparsity and modularity priors as regularizers, respectively. We apply our proposed methods to identify subjects with MCI and ASD from their corresponding NCs. In both MCI and ASD identifications, our proposed HoFCN methods consistently outperform the baseline methods. Especially, the M-HoFCN tends to achieve the best performance, which illustrates the importance of the modularity prior in FCN estimation.

## Author Contributions

DS put forward the idea of HoFCN and provided the preprocessed rs-fMRI data. LQ and YZ proposed to learn the neighborhood networks that meet sparsity and modularity for improved HoFCN estimation. YZ, LZ, and ST designed the procedures of MCI/ASD identification experiments. All authors developed the estimation algorithm and contributed to the preparation of the article, figures, and tables.

### Conflict of Interest Statement

The authors declare that the research was conducted in the absence of any commercial or financial relationships that could be construed as a potential conflict of interest.
